# A Genome-Wide Association Study Identifies New Loci Involved in Wound-Induced Lateral Root Formation in *Arabidopsis thaliana*

**DOI:** 10.3389/fpls.2019.00311

**Published:** 2019-03-15

**Authors:** María Salud Justamante, Sergio Ibáñez, Adrián Peidró, José Manuel Pérez-Pérez

**Affiliations:** ^1^Instituto de Bioingeniería, Universidad Miguel Hernández de Elche, Elche, Spain; ^2^Departamento de Ingeniería de Sistemas y Automatización, Universidad Miguel Hernández de Elche, Elche, Spain

**Keywords:** wound-induced lateral root formation, root system architecture, genome-wide association mapping, single nucleotide polymorphism, natural variation, histidine phosphotransfer protein

## Abstract

Root systems can display variable architectures that contribute to nutrient foraging or to increase the tolerance of abiotic stress conditions. Root tip excision promotes the developmental progression of previously specified lateral root (LR) founder cells, which allows to easily measuring the branching capacity of a given root as regards its genotype and/or growth conditions. Here, we describe the natural variation among 120 *Arabidopsis thaliana* accessions in root system architecture (RSA) after root tip excision. Wound-induced changes in RSA were associated with 19 genomic loci using genome-wide association mapping. Three candidate loci associated with wound-induced LR formation were investigated. Sequence variation in the hypothetical protein encoded by the At4g01090 gene affected wound-induced LR development and its loss-of-function mutants displayed a reduced number of LRs after root tip excision. Changes in a histidine phosphotransfer protein putatively involved in cytokinin signaling were significantly associated with LR number variation after root tip excision. Our results provide a better understanding of some of the genetic components involved in LR capacity variation among accessions.

## Introduction

Strong modulation of root system architecture (RSA) by environmental cues, such as nutrient and water availability has been a well-documented process in *Arabidopsis thaliana* (Giehl and von Wirén, 2014; Giehl et al., 2014; Robbins and Dinneny, 2015). In primary roots (PRs) (PRs), a regular pre-branching pattern of lateral roots (LRs) is established by an endogenous periodic oscillation in gene expression near the root tip (Moreno-Risueno et al., 2010). A subset of xylem pole pericycle cells within the pre-branch sites becomes specified as LR founder cells. Subsequently, LR founder cells undergo a self-organizing and non-deterministic cell division patterning (Lucas et al., 2013; von Wangenheim et al., 2016) to initiate a LR primordium that eventually emerges through the PR tissues (Peret et al., 2009; Du and Scheres, 2018). However, the developmental progression of individual LR primordia is dependent on environmental cues, such as water distribution within the soil (Bao et al., 2014). In addition, a local auxin source from the LR cap of the PR, which is derived from the auxin precursor indole-3-butyric acid (IBA), determines whether a pre-branch site is specified or not (Xuan et al., 2015). The spatial distribution of LRs is not fixed, yet the total number of LR competent sites was stable with time. Root tip excision promotes the developmental progression of nearly all pre-branch sites toward LR emergence, providing an accurate measure for LR branching capacity. This later approach will allow assessing whether changes in LR pre-patterning have occurred in different genotypes and/or growth conditions (Van Norman et al., 2014). These results are in agreement with the current view that cells at the root tip are capable of integrating information about the local soil environment, tailoring the RSA for optimal nutrient and water uptake or after PR damage (Robbins and Dinneny, 2015).

Genome-wide association (GWA) studies have contributed to the identification of natural variation in key genes controlling PR growth under control and abiotic stress conditions (Meijon et al., 2014; Slovak et al., 2014; Satbhai et al., 2017; Bouain et al., 2018). Natural variation in RSA has also been reported (Rosas et al., 2013). Salt-induced changes in RSA were associated with more than 100 genetic loci identified by GWA mapping, some of which are involved in ethylene and abscisic acid (ABA) signaling (Julkowska et al., 2017). In addition, strong additive effects of phosphate starvation on LR density and salt stress on LR length were found in a recent study with a large number of Arabidopsis accessions (Kawa et al., 2016). Their results suggested that the integration of signals from phosphate starvation and salt stress might partially rely on endogenous ABA signaling. One of the candidate genes identified in these studies was *HIGH-AFFINITY K^+^ TRANSPORTER1* (*HKT1*), previously identified for its role in salinity tolerance by modulating sodium/potassium homeostasis (Munns et al., 2012). The targeted HKT1 expression to pericycle cells reduced LR formation under salt stress (Julkowska et al., 2017). Recently, Ristova et al. (2018) reported a comprehensive atlas of RSA variation upon treatment with auxin, cytokinin (CK) and ABA in a large number of *A. thaliana* accessions. In that study, hierarchical clustering analyses identified groups of accessions sharing similar or diverse responses to a particular hormone perturbation that can be very useful to identify accessions that behave differently than the bulk and to use them as parents for QTL mapping.

To explore the natural variation of LR branching capacity in Arabidopsis (Van Norman et al., 2014), we performed a wound-induced LR formation assay in 174 accessions from the Haplotype Map (HapMap) collection (Weigel and Mott, 2009). GWA mapping using data from 120 accessions revealed 162 SNP associations with several RSA traits measured after root tip excision. SNPs affecting six genes were found significantly associated with LR number variation.

## Materials and Methods

### Plant Materials and Growth Conditions

Our population for GWA mapping consisted of 174 natural inbred lines (i.e., accessions) of *A. thaliana* (L.) Heyhn. selected from the 1001 Genomes Project (Weigel and Mott, 2009) based on marker information and seed availability (Supplementary Table S1). The laboratory strain Columbia-0 (Col-0) was chosen as the reference. The following lines (in the Col-0 background) were used to isolate T-DNA homozygous mutants of the studied genes: N572850, N586312, N616200, and N620707 (Supplementary Table S2). The *ahp1 ahp2 ahp3* (Hutchison et al., 2006) mutants were also used. All lines used were obtained from the Nottingham Arabidopsis Stock Centre (NASC^1^). Seeds were stored at 4°C for several weeks (>12) to break dormancy.

Seeds were surface-sterilized in 2% (w/v) NaClO and rinsed with sterile water before being transferred to 120 mm × 120 mm × 10 mm Petri dishes containing 75 mL of one-half-Murashige & Skoog (MS) medium with 2% sucrose, 8 g/L plant agar (Duchefa Biochemie, Netherlands) and 1× Gamborg B5 vitamin mixture (Duchefa Biochemie). After 4 days of stratification at 4°C in darkness, plates were wrapped in aluminum foil and were transferred (0 days after sowing) to an MLR-352-PE growth chamber (Panasonic, Japan) at 22 ± 1°C during 3 days in a nearly vertical position. Plates were unwrapped (3 days after sowing) and grew during another 3 days with continuous light (50 μmol⋅m^−2^⋅s^−1^). For each accession, 12 seeds were sown per petri dish in triplicate (36 samples/line). Sixteen consecutive sowings including 11 accessions each were established. Additionally, Col-0 was also included in all the sowings to be used as the growth reference accession and for normalization purposes (Supplementary Figure S1A). Lines with germination percentage lower than 80% and with ambiguous marker information were discarded for further analysis (Supplementary Table S1).

### Induction of Lateral Root Formation

**FIGURE 1:**
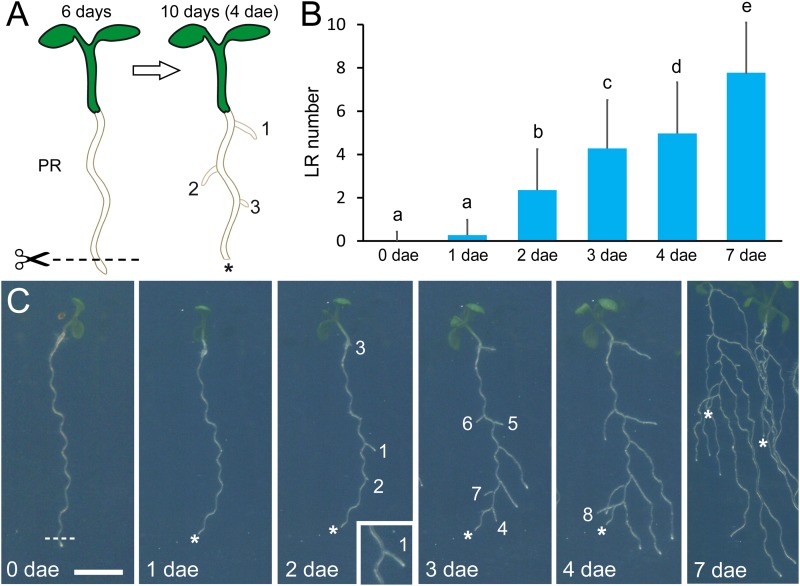
Wound-induced lateral root (LR) development. **(A)** Schematic representation of LR induction by root tip excision (the excision site is indicated by the scissors). dae, days after excision; PR, primary root. **(B)** Average LR number observed in the Columbia-0 (Col-0) reference accession after root tip excision. Different letters indicate significant differences (LSD, *P*-value < 0.01). **(C)** Time course of LR development in Col-0 after root tip excision. Scale bar: 5 mm. Numbers in **(A,C)** indicate order of LR emergence (1, first; 2, second; etc.). Asterisks mark the PR tips after excision.

To induce LR development during early seedling growth (Van Norman et al., 2014), we excised about 2 mm of the root tip using a sterile scalpel on a laminar flow hood at 6 days after sowing (Figure 1A). Next, samples were transferred back to the growth chamber and followed during 4 days for the analysis of several root traits as described below.

### Image Processing and Parameter Measurement

Petri dishes were daily imaged from 6 to 10 days after sowing using an Epson Perfection V330 Photo scanner (Seiko Epson Corporation, Nagano, Japan), at a resolution of 600 dpi, and saved as RGB color images in JPEG file format. Scanned images were processed using EZ-Rhizo (Armengaud et al., 2009) with available plug-in macros to convert them into binary images, remove noise, gap-filling, and skeletonize them prior to automated root detection (Supplementary Figures S1B–S1E). PR length was directly obtained from the EZ-Rhizo output files from scanned images of 6 days after sowing. A highly significant and positive correlation was found for PR length estimated by EZ-Rhizo and directly measured by ImageJ software^2^ from hand-drawn roots (Supplementary Figure S1F). LR number was visually counted from the scanned images between 6 and 10 days after sowing. LR emergence onset corresponds to the day when the first newly emerged LR was visible. LR density was estimated at 8 and 10 days after sowing by the LR number/PR length ratio. Data values were normalized relative to Col-0 values in each sowing dividing each individual value by the Col-0 average (Supplementary Table S3).

### Statistical Analyses and Heritability Estimation

Statistical analyses of the data was performed by using StatGraphics Centurion XV (StatPoint Technologies, United States) and SPSS 21.0.0 (SPSS Inc., United States) software packages. Data outliers were identified based on aberrant standard deviation values and were excluded for posterior analyses as described elsewhere (Aguinis et al., 2013). One-sample Kolmogorov–Smirnov tests were performed to analyze the goodness-of-fit between the distribution of the data and a theoretical normal distribution. Non-parametric tests and data transformation were applied when needed. To compare the data for a given variable, we performed multiple testing analyses with the ANOVA F-test or the Fisher’s LSD (Least Significant Differences) methods. Significant differences were collected with 5% level of significance (*P*-value < 0.05) unless otherwise indicated.

The broad-sense heritability (*H^2^*) for the studied dataset was calculated as *H^2^* = σ_G_^2^/(σ_G_^2^ + σ_GE_^2^/e +σ^2^_*e*_2/re), in which σ_G_^2^, σ_GE_^2^, σ_*e*_^2^, r, and e represent the estimated variances for the genetic effects, genotype-environment interactions, random errors, number of replications (12) and number of environments (three), respectively. The estimated variances for σ_G_^2^, σ_GE_^2^, and σ_*e*_^2^ were obtained by ANOVA using normalized data values as regards to the Col-0 reference accession from 106 of the studied lines.

### Population Structure Analysis

The population structure of the selected accessions was estimated using the Bayesian model-based clustering algorithm (Porras-Hurtado et al., 2013) implemented in Structure v2.3.4 software^3^ (Falush et al., 2003). To this end, we used a collection of 319 randomly selected bi-allelic synonymous (likely evolutionary-neutral) SNP markers from available sequence data (Atwell et al., 2010; Cao et al., 2011; Seren et al., 2012). An in-house Matlab script (Supplementary Table S4) was used for single nucleotide polymorphism (SNP) data selection and file formatting. Structure analysis was performed for *K* = 1 to *K* = 10 clusters with 20 replicates and 50,000 burn-in period iterations, followed by 50,000 Markov chain Monte Carlo iterations while using a population admixture ancestry model. To determine the most likely number of subpopulations (*K*) we applied the Δ*K* method, as described elsewhere (Evanno et al., 2005).

### Genome-Wide Association Studies

Genome-wide association mapping was performed using the GWAPP web interface^4^, which contains genotypic information for up to 250,000 bi-allelic SNP markers (Seren et al., 2012). GWAS was conducted for the studied traits using the linear regression model (LM) to identify associations between the phenotype of 120 studied accessions and the 205,978 SNPs available in the database. Relative LR numbers were transformed using the *y* = $\sqrt{x}$ function to fit the theoretical normal distribution. Association mapping was obtained excluding from the analyses all SNPs with a frequency <0.12. SNPs with a −log_10_(*P*-value) > 6.5 were considered significantly associated to the studied trait (Supplementary Table S5). Manhattan plots, representing the genomic position of each SNP and its association [−log_10_(*P*-value)] with the studied trait, were downloaded from the GWAPP web interface. We analyzed the sampling bias on GWAS by systematically removing one or several geographically isolated accessions and found that it did not make any difference to the detected SNP associations (Supplementary Table S6). We selected non-synonymous SNPs with a −log_10_(*P*-value) > 6.5 (*P*-value = 3.16 × 10^−7^) for further studies.

### Genotyping

Seedlings with T-DNA homozygous insertions in the annotated genes were identified by PCR verification with T-DNA specific primers and a pair of gene-specific primers (Supplementary Table S2). Genomic DNA isolation and genotyping of T-DNA insertion loci PCR were performed as described elsewhere (Pérez-Pérez et al., 2004).

### Gene Expression Analysis by Real-Time Quantitative PCR

Primers amplified 81–178 bp of the cDNA sequences (Supplementary Table S2). To avoid amplifying genomic DNA, forward and reverse primers bound different exons and hybridized across consecutive exons.

RNA extraction and cDNA synthesis were performed as described elsewhere (Villacorta-Martín et al., 2015). For real-time quantitative PCR, 14 μl reactions were prepared with 7 μl of the SsoAdvanced Universal SYBR Green Supermix (Bio-Rad, United States), 4 μM of specific primer pairs, and 1 μl of cDNA- and DNase-free water (up to 14 μl of total volume reaction). PCR amplifications were carried out in 96-well optical reaction plates on a Step One Plus Real-Time PCR System (Applied Biosystems, United States). Three biological and two technical replicates were performed for each gene. The thermal cycling program started with a step of 10 s at 95°C, followed by 40 cycles (15 s at 95°C and 60 s at 60°C), and the melt curve (from 60 to 95°C, with increments of 0.3°C every 5 s). Dissociation kinetics of the amplified products confirmed their specificity.

Primer validation and gene expression analyses were performed by the absolute quantification method (Lu et al., 2012) by using a standard curve that comprised equal amounts from each cDNA sample. The housekeeping At4g26410 gene (*RGS1-HXK1 INTERACTING PROTEIN 1*, *RHIP1*) (Czechowski et al., 2005) was chosen as an internal control and to ensure reproducibility. In each gene, the mean of fold-change values relative to the Col-0 reference genotype was used for graphic representation. Relative expression values were analyzed using SPSS 21.0.0 (SPSS Inc., United States) by applying the Mann–Whitney U-test for statistical differences between cDNA samples (*P*-value < 0.05).

## Results

### Natural Variation of Wound-Induced Lateral Root Formation

To validate our experimental approach (Figure 1A), we studied wound-induced LR formation in Columbia-0 (Col-0) during 7 days. PR length remained almost invariable after root tip excision during the whole experiment (Supplementary Figure S1G). The new LRs were already visible at 1 day after PR tip excision (1 dae) and reached 4.97 ± 2.36 (*n* = 467) LRs at 4 dae (Figure 1B). At 7 dae, the number of LRs slightly increased but it was not possible to measure it unambiguously due to overlap between the LRs of adjacent seedlings (Figure 1B,C). We did not observe a clear spatial pattern of LR emergence from the PRs except that, in all cases, the new LRs emerged from its convex side (Figure 1C, inset). We found a slight variation in PR length and LR number between the different sowings (Supplementary Figures S2A,B), which might be caused by subtle environmental differences at the growth chamber.

**FIGURE 2:**
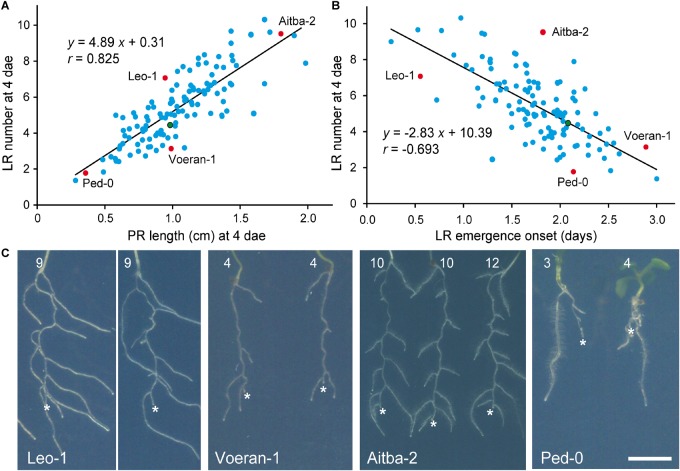
Phenotypic variation of LR architecture after root tip excision. **(A,B)** Scatter plots of average values for LR number and PR length, or LR number and LR emergence onset among the studied accessions. **(C)** Some accessions with extreme LR architecture phenotypes after root tip excision. Asterisk marks the PR tip after excision and numbers refer to LR number at 4 dae. Scale bar: 5 mm. Red dots indicate the accessions with contrasting phenotypes shown in **(C)** and green dots correspond to Col-0.

We studied wound-induced LR formation in a collection of 173 additional accessions selected from the 1001 Genomes Project (Weigel and Mott, 2009; Supplementary Table S1). In 34 of the studied accessions, the germination percentage at 6 days after sowing was lower than 80% and were discarded for further analysis; other 20 accessions were also discarded because of ambiguous genotypes at the GWAPP web interface (Seren et al., 2012) (Supplementary Table S1). We found variation in all the studied traits (Supplementary Figure S2B). Exceptionally, one or two LRs were observed before root tip excision (0 dae) in some samples, but these were not considered. The broad-sense heritability (*H*^2^) was calculated for each of the studied traits (see section “Materials and Methods”). Heritability estimates ranged between 0.90 (LR emergence onset and LR density) and 0.95 (LR number). Broad-sense heritability for PR length was 0.93. Interestingly, we found a positive and significant correlation between LR number and PR length at 4 dae (*r* = 0.83; Figure 2A), as well as a negative and significant correlation between LR number at 4 dae and LR emergence onset (*r* = −0.69; Figure 2B). LR number ranged from 1.38 ± 0.82 in Ru3.1-31 (PR length: 0.28 ± 0.07 cm; *n* = 24) and 9.42 ± 4.84 in Kidr-1 (PR length: 1.90 ± 0.50 cm; *n* = 24). Hence, reduced LR number in Ru3.1-31 compared to Kidr-1 was likely caused by its reduced PR length. Some accessions, such as Leo-1 and Voeran-1 displayed contrasting phenotypes as regards their LR number (7.07 ± 1.41 and 3.14 ± 1.48 LRs, respectively; *n* = 29) although they displayed similar PR lengths (Figure 2A,C). On the other hand, Leo-1 and Aitba-2 displayed similar LR number which were larger in Leo-1 likely due to its earlier LR emergence onset (0.55 ± 0.57 days in Leo-1 and 1.82 ± 0.50 days in Aitba-2; *n* = 29; Figure 2B,C). Ped-0 displayed very short PRs while their wound-induced LRs were much longer (Figure 2C). As regards LR density, Castelfed4.2 and Leo-1 displayed extreme phenotypes, with 2.98 ± 1.35 and 8.05 ± 2.37 roots/cm (*n* = 29), respectively.

### Assessment of Population Structure

**FIGURE 3:**
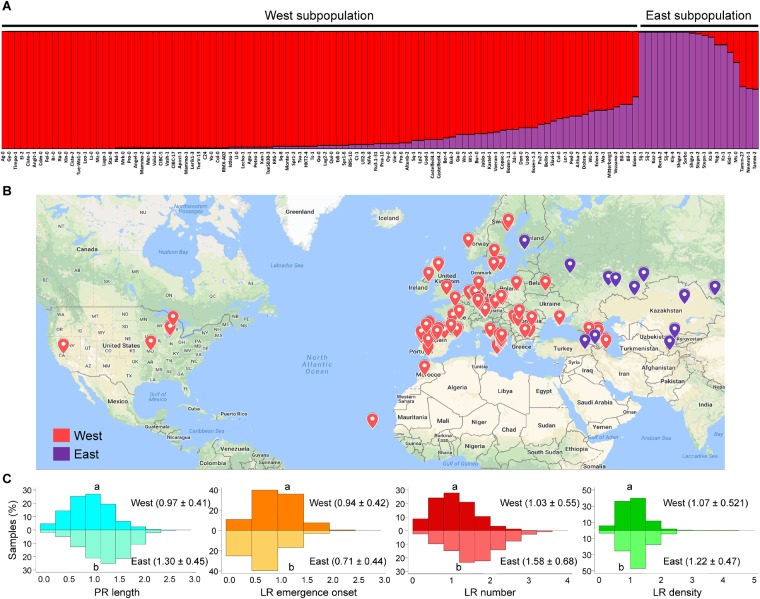
Population structure of the studied accessions. **(A)** Population structure for the studied accessions using a collection of 319 whole-genome distributed and randomly selected synonymous single nucleotide polymorphism (SNP) markers. **(B)** Geographical localization of the studied accessions. Accessions from the “West” subpopulation are labeled in red; those from the “East” subpopulation are labeled in purple. **(C)** Histograms for the studied parameters. Relative data values at 4 dae as compared with the Col-0 reference accession from the “West” subpopulation are represented in the top histograms, while those from the “East” subpopulation are represented in the bottom histograms. Different letters indicate significant differences (LSD, *P*-value < 0.05).

The observed phenotypic distribution for the studied traits (PR length, LR emergence onset, LR number and LR density) suggested that these traits were controlled by multiple genes, that some of the causal alleles are pleiotropic (i.e., affect several of these traits), and that the studied population (*n* = 120 accessions) was polymorphic for those causal alleles. We determined the genetic relationship among the studied accessions using a Structure analysis with 319 genome-wide randomly selected and synonymous (likely evolutionary neutral) SNP markers already available (see section “Materials and Methods”). Structure analysis of these accessions identified two distinct genetic groups (Figure 3A) that closely correspond to their geographic regions of origin (Figure 3B): The so-called “West” subpopulation including 101 accessions, and the “East” subpopulation with the remaining 19 accessions. However, a detailed analysis of these results indicated a continuous genetic shift from “East” to “West” accessions that follow *A. thaliana* geographical distribution and that likely arose by local haplotypes, as it has been previously proposed (Platt et al., 2010).

**FIGURE 4:**
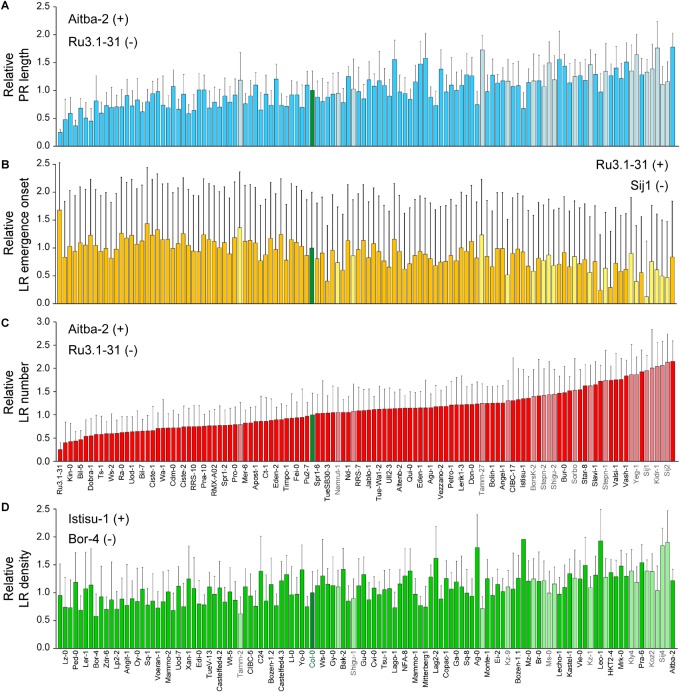
Natural variation of LR architecture after root tip excision in 120 accessions of *Arabidopsis thaliana*. Average ± standard deviation (SD) values of **(A)** relative PR length, **(B)** relative LR emergence onset, **(C)** relative LR number, and **(D)** relative LR density at 4 dae as compared with the Col-0 reference accession. Light-colored bars indicate accessions belonging to the “East” subpopulation (names indicated in gray). The Col-0 reference is shown in green. Accessions are sorted based on their relative LR numbers. Those accessions with extreme phenotypes (+, maximum; −, minimum) are also indicated.

We found a significant variation range for the studied traits between these two genetically distinct subpopulations (Figure 3C). Altogether, accessions belonging to the “East” subpopulation displayed longer PRs and an increasing number of LRs as regards the “West” subpopulation. However, some accessions of the “East” subpopulation, such as Shigu-1, displayed lower phenotypic values for the studied traits than most of their relatives (Figure 4). On the other hand, accessions of the “West” subpopulation and highly genetically divergent from those in the “East” subpopulation (i.e., Aitba-2, HKT2-4, Leo-1, Mrk-0, and Pra-6) displayed higher number of LRs compared with their closest relatives (Figure 4). Despite some population structure among the studied lines and due to high heritability estimates, there is potential for the identification of natural alleles affecting wound-induced LR formation responses through GWA mapping with our dataset.

### Genome-Wide Association Mapping of Wound-Induced LR Formation

To obtain insight into the genetic basis of the observed variation in wound-induced responses in young *A. thaliana* roots, we performed GWA mapping (see section “Materials and Methods”). The significance of association was first evaluated with three different statistical models (LM, KW and AMM; Supplementary Figures S3A–C) and no significant SNP associations were identified by randomization of the phenotypic values within the studied lines (Supplementary Figure S3D). Although LM, and KW usually include more false positives than AMM, they do not present any risk of overcorrection in *P*-value when applied to traits correlated with population structure (Filiault and Maloof, 2012). We used a conservative threshold of −log_10_(*P*-value) > 6.5 and minor allele frequency (MAF) > 12% to select the SNPs being associated with a given trait. A total of 162 SNP associations were found with the LM method for the studied parameters (Supplementary Table S5). We found 32 SNPs associated with PR length with *P*-values ranging from 1.41 × 10^−10^ to 3.04 × 10^−7^. Thirty-two SNPs were significantly associated with LR emergence onset (*P*-values ranging from 1.22 × 10^−8^ to 3.09 × 10^−7^) and only one SNP was found associated with LR density. The larger number of significantly associated SNPs was found for LR number (*n* = 114), with *P*-values ranging from 8.27 × 10^−11^ to 3.15 × 10^−7^. Consistently with our previous observation that PR length and LR number are significantly correlated, 11 SNPs were significantly associated with both traits; similarly, six significantly associated SNPs were shared between LR emergence onset and LR number (Supplementary Figure S4A).

**FIGURE 5:**
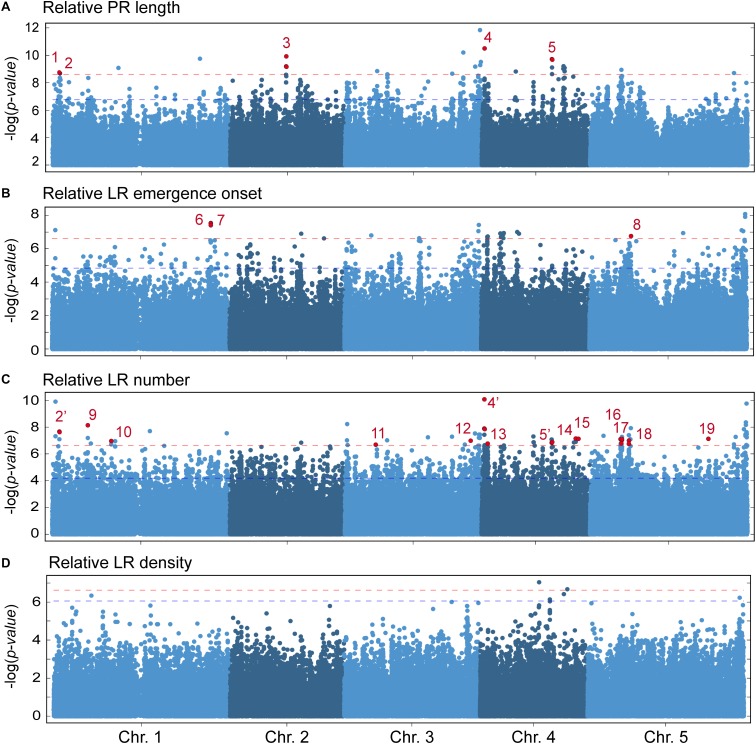
Manhattan plots of associations between SNPs and the studied parameters using a linear regression model (LM). **(A)** Relative PR length, **(B)** relative LR emergence onset, **(C)** relative LR number, and **(D)** relative LR density at 4 dae as compared with the Col-0 reference accession. Dashed horizontal red lines indicate threshold for significance in genome-wide association (GWA) mapping set at –log_10_(*P*-value) > 6.5. Red dots indicate the position of non-synonymous significantly associated SNPs. Numbers 1–19 indicate the genomic regions considered for further studies. Some statistically significant SNP were found both in PR length and LR number (2 and 2′, 4 and 4′, 5 and 5′).

Next, we classified the selected SNPs based on its molecular effect (Supplementary Figure S4B). About 36% of the significantly associated SNPs were located in intergenic regions and 17.2% of the SNPs laid in the coding region of the annotated gene causing amino acid changes in the protein (Supplementary Figure S4B). Previous reports have shown that, due to linkage disequilibrium, multiple significantly associated SNPs should be found within a small chromosome region for true associations (Rajarammohan et al., 2018). To reduce the number of selected loci for further studies, we focused on 19 candidate genomic regions based on the following criteria (Supplementary Figure S4C): (1) *P*-value of associated SNPs < 3.16 × 10^−7^ (which corresponded to a LOD score > 6.5), (2) presence of multiple significantly associated SNPs within an average of 10 Kpb genomic window, and (3) presence of, at least, one non-synonymous SNP within the selected region. We found five genomic regions putatively contributing to PR length variation in the studied population (Figure 5A), with one (At1g04260), two (At1g04470), three (At2g22660), one (At4g01090), and two (At4g22920, At4g22940) non-synonymous SNPs each (Supplementary Table S5). Three genomic regions were identified as regards their effect on variation in LR emergence onset (Figure 5B), each with one non-synonymous SNP (At1g72250, At1g72300 and At5g20980, respectively; Supplementary Table S5). We found 14 genomic regions putatively involved in the observed variation in LR number among the studied accessions (Figure 5C). Interestingly, the non-synonymous SNPs of three of these regions (dubbed as 2′, 4′, and 5′) overlapped with three genomic regions also selected as being involved in PR length variation (Supplementary Table S5). Hence, the affected genes in these cases, At1g04470, At4g01090, and At4g22940, might indirectly contribute to the LR number differences likely due to their direct effect on PR length before root tip excision. The remaining regions identified in the GWA analysis for LR number might include genes that directly contribute to wound-induced LR formation (Supplementary Table S5) and therefore deserve further studies. On the contrary, no other genomic region fulfilled our selection criteria as regards LR density and this trait was not considered (Figure 5D).

**Table 1:** Candidate genes for LR number identified in the genome-wide association (GWA) mapping.

Gene	SNP coordinate	-log(*P*-value)	Description (domain/name)	Root expression^a^	Reference
At1g04470	Chr1:1214221	7.660	Hypothetical protein (DUF810)	N/A	Hanada et al., 2011
	Chr1:1214425	7.660			
At1g17700	Chr1:6089805	8.130	Prenylated RAB acceptor 1.F1	Elongation zone (EZ) and mature endodermis	Alvim Kamei et al., 2008
At3g58220	Chr3:21566092	6.980	TRAF-like family protein/ MATH domain RTM3-like protein	N/A	N/A
At4g01090	Chr4:472438	7.876	Hypothetical protein (DUF3133)	EZ and LR primordium	Ascencio-Ibáñez et al., 2008
	Chr4:472726	10.082			
	Chr4:473027	7.889			
At4g33530	Chr4:16128906	7.143	Potassium transporter (KUP5)	EZ and mature cortex	Ahn et al., 2004
At5g16220	Chr5:5299807	7.080	Octicosapeptide/Phox/Bem1p family protein	Columella stem cells	N/A
At5g19710	Chr5:6665363	7.000	Histidine containing phosphotransfer protein	Lateral root cap	Nishizawa et al., 2006
At5g49880	Chr5:20286041	7.129	Spindle assembly checkpoint protein (MAD1)	Meristem and LR primordium	Du and Scheres, 2018

*^a^Gene expression was obtained from the eFP browser (Winter et al., 2007). N/A: not available.*

### Selection of Candidate Genes Involved in Wound-Induced LR Formation

To identify allelic variation in genes contributing to the observed differences in LR number after root tip excision, we selected 20 non-synonymous SNPs for further studies (Supplementary Table S6). Although SNPs in intergenic regions could also be causative, we decided to focus on non-synonymous polymorphisms as their later characterization can be performed on an easier way using reverse genetics tools. Based on both geographic distribution and genotype (Figure 3), we hypothesized that Nemrut-1 and Yeg-1 might represent atypical accessions due to ancient migration and genetic isolation. To discard the false-positive SNPs of this spurious association, we repeated the GWA mapping by removing either one or both accessions, which allowed us to reduce to 11 the number of significantly associated SNPs contributing to LR number (Supplementary Table S6). Due to the genetic structure of the studied accessions, we performed ANOVA analyses as regards the “East” and “West” subpopulations independently. Polymorphisms at eight SNPs affecting six genes (At1g17700, At3g58220, At4g01090, At4g33530, At5g16220, and At5g19710; Table 1) were found significantly associated with LR number variation (Supplementary Table S6). Four haplotypes were detected for selected SNPs within the At4g01090 gene (CAA, CAT, CTA, and TAT). The accessions containing the TAT haplotype displayed a significant increase in LR number, irrespectively of their subpopulation of origin (Supplementary Figure S5A). Indeed, the T to A polymorphism at Chr4:472726 accounted for the quantitative differences in LR number by its own. In addition, we observed a haplotype-dependent relationship between SNPs at At5g16220, and At5g19710, which are separated by 1.4 Mb and were previously assigned to two different candidate genomic regions (Figure 5C). The GA haplotype for these two genes corresponded to the higher phenotypic values for LR number (Supplementary Figure S5B). To our knowledge, this is the first example of two-linked quantitative trait nucleotides (QTNs) detected through GWA mapping and further experiments will account for the functional relationship between the two genes and wound-induced LR number.

### Experimental Validation of Candidate Genes

To validate the identification of novel genes involved in wound-induced LR formation in *A. thaliana* seedlings, we chose At4g01090, At4g33530, and At5g19710 for further studies. We searched for available T-DNA insertions in those three genes and identified homozygous mutants by means of PCR and sequencing (Supplementary Table S1). Based on haplotype studies, we found that the Chr4:472726 C/T polymorphism at the At4g01090 gene (Supplementary Figure S6A) was significantly associated with LR number variation, even in those accessions belonging to the same subpopulation such as Fei-0 (5.19 ± 1.88; *n* = 36) and Star-8 (8.36 ± 2.33; *n* = 36; Supplementary Figures S6B,C). Additionally, T-DNA homozygotes from the Salk_086312 segregating line displayed a reduced number of wound-induced LR at 4 dae (2.82 ± 1.27; *n* = 34) in comparison to their wild-type siblings (6.68 ± 1.87; *n* = 63; Supplementary Figures 6D,E). The homozygous seedlings were also characterized by their longer hypocotyl and shorter PRs (Supplementary Figure 6D). Our results confirmed that the hypothetical protein encoded by the At4g01090 gene participates in wound-induced LR development and that the observed natural variation in their protein sequence might affect their biochemical activity.

At4g33530 (Supplementary Figure S7A) encodes a potassium (K^+^) uptake transporter which is highly expressed in root hairs (Ahn et al., 2004). We found a statistically significant and non-synonymous SNP (Chr4:16128906) correlated with wound-induced LR phenotype variation (Supplementary Figure S7B). The accessions Pu2-7 and Aitba-2 differed in their LR number (6.44 ± 1.95; *n* = 32 and 9.53 ± 1.94; *n* = 34, respectively) and carried alternative alleles of the Chr4:16128906 marker (Supplementary Figure S7C). We identified T-DNA homozygotes from two Salk insertion lines interrupting the coding region of this gene (Supplementary Figure S7A). None of the studied homozygous mutants from Salk_120707 and Salk_072850 lines displayed significant differences in wound-induced LR number as regards their wild-type siblings (Supplementary Figures S7D,E).

**FIGURE 6:**
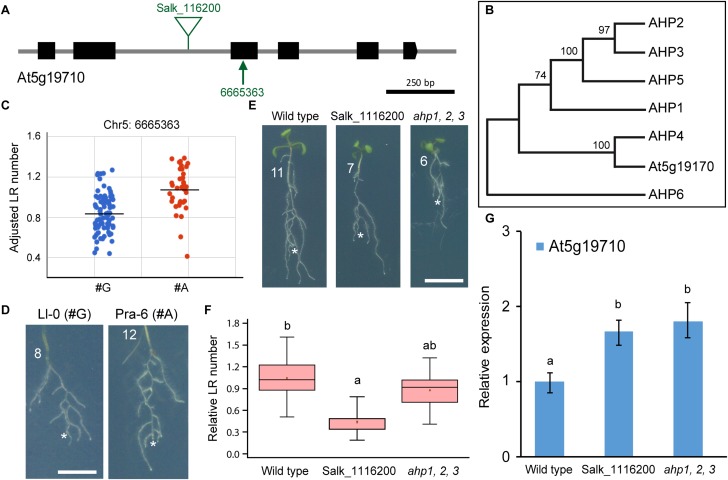
Functional analysis of At5g19710 variation concerning LR number. **(A)** Gene structure of At5g19710. Exons are represented by black boxes and introns are depicted as gray lines. The studied non-synonymous SNP markers (arrows) and annotated T-DNA insertions (triangles) are indicated. **(B)** Phylogenetic analyses of the AHP gene family. Trees were drawn to scale, with branch lengths representing the number of substitutions per site. These analyses were conducted in MEGA7 as described elsewhere (Sánchez-García et al., 2018). **(C)** Scatterplot of average LR number values used for GWA mapping, sorted by the alleles, G and A, at Chr5:6665363 position. **(D)** Some accessions of the studied haplotypes with contrasting LR number values. **(E)** Representative images of wild-type and homozygous seedlings of indicated Salk and mutant lines at 4 dae. Asterisk marks the PR tip after excision and numbers refer to LR number at 4 dae. Scale bars: 5 mm. **(F)** Boxplot showing relative LR number values of wild type and homozygous T-DNA mutants of the indicated Salk lines. Different letters indicate significant differences (LSD, *P*-value < 0.01). (**G**) RT-qPCR of the expression of At5g19710 in wild-type and mutant seedlings at 7 dae. Bars indicate normalized expression levels ± standard deviation relative to the wild type. Asterisks indicate significant differences between the assayed genotypes and the reference (LSD; *P*-value < 0.05).

The At5g19710 gene (Figure 6A) encodes a histidine phosphotransfer protein (AHP) whose function on the CK transduction pathway has not yet been elucidated. There are six other known AHPs involved in CK responses (Hutchison et al., 2006). Phylogenetic tree reconstruction of AHPs including At5g19710 (Figure 6A), suggested that the annotated AHP protein encoded by this gene was incorrectly predicted due to an exon skipping, and clustered together with the AHP4 negative regulator of CK signaling (Figure 6B; Moreira et al., 2013). We found that the Chr5:6665363 G/A polymorphism at the third exon of this gene (Figure 6A) was significantly associated with LR number variation (Figure 6C), even in those accessions belonging to the same subpopulation such as Ll-0 (3.91 ± 2.20; *n* = 32) and Pra-6 (7.91 ± 1.91; *n* = 33; Figure 6D). We identified a homozygous T-DNA insertion line for the At5g19710 gene whose seedlings showed a reduced number of wound-induced LRs (Figure 6E,F) due to a significant miss-regulation of At5g19710 gene expression (Figure 6G). We also confirmed that the triple *ahp1 ahp2 ahp3* mutants, which was defective in CK root responses (Hutchison et al., 2006), displayed a significant reduction in wound-induced LRs at 4 dae compared to their wild-type background (Figure 6E,F). Taken together, our results seem to indicate that altered homeostasis of AHP proteins required for CK signaling interferes with wound-induced LR formation, a statement that requires further investigation.

Finally, we wondered whether there was an epistatic interaction between the allelic variants for some of the studied non-synonymous SNP markers (Chr4:472726, Chr4:16128906 and Chr5:6665363, respectively) that contributed to the observed variation in wound-induced LR formation in the studied population. Accessions sharing the CCG haplotype for these three markers displayed the smallest number of wound-induced LRs (Supplementary Figure S8A), while only the accessions with the haplotype containing a single polymorphism in the At4g01090 gene showed a significant increase (LSD; *P*-value < 0.01) in LR numbers (Supplementary Figure S8A). The individual contribution of the SNP polymorphisms in the other two genes considered, At4g33530 and At5g19710, hardly increased wound-induced LR numbers alone but in combination (CGA haplotype) their effects on wound-induced LR formation were enhanced (Supplementary Figure S8A). Similar interactions were found between the other SNP pairs (TGG and TCA haplotypes). Interestingly, we identified several accessions in the “West” subpopulation of all the haplotypes correlated with an increase in wound-induced LR numbers, such as Mrk-0 (8.17 ± 2.12; *n* = 36) and Vie-0 (6.33 ± 1.80; *n* = 33) (Supplementary Figure S8B). However, all accessions with the TGA haplotype combining the allelic variants that contribute to an increase of wound-induced LR formation belonged to the “East” subpopulation, which suggested that this trait might be ancestral.

## Discussion

The spatial configuration of the RSA allows the plant to dynamically respond to changing soil conditions (Koevoets et al., 2016). Root plasticity relies on the integration of systemic and local signals of nutrient and water availability into the core developmental program of the root (Araya et al., 2014; Bao et al., 2014). Periodic fluctuations in auxin response within the vascular region of the PR near the meristem control the patterning of LR founder cell specification (Moreno-Risueno et al., 2010). A novel IBA-to-IAA conversion pathway in the outer LR cap cells creates a local auxin source that contributes to these periodic auxin fluctuations, which in turn are essential for LR pre-patterning (Xuan et al., 2015). Our wound-induced RSA assay is simple and provides an accurate measure of LR capacity, defined as the total number of competent sites for LR formation on a given root (Van Norman et al., 2014). Interestingly, a recent study has demonstrated that *A. thaliana* PRs might use a specific pathway to activate LR formation when the PR is damaged (Sheng et al., 2017). The authors found that *WUSCHEL-related homeobox11* (*WOX11*), which is involved in *de novo* regeneration of adventitious roots from leaf explants (Liu et al., 2014), was also required for LR formation in soil conditions, likely upstream of the LATERAL ORGAN BOUNDARIES DOMAIN (LBD) genes required for LR initiation (Goh et al., 2012).

We found a wide variation for the studied wound-induced RSA traits in our GWA mapping population. There was a clear correlation between the number of wound-induced LRs (i.e., LR branching capacity) with PR length at the excision day and with the time of LR emergence after excision. Some accessions, such as Leo-1 and Voeran-1, significantly differed in their LR number because of an earlier initiation of wound-induced LRs in Leo-1, which were also longer. Hence, Leo-1 contained alleles for higher LR branching capacity that positively contributed to an enhanced root system, which might allow survival in harsh environments. It will be interesting to evaluate whether differences in *WOX11* expression between these accessions might account for the observed differences in RSA after root tip excision.

A population analysis of the studied accessions inferred two distinct genetic groups that closely corresponded to the geographic regions of *A. thaliana* native distribution and the proposed postglacial colonization routes in this species (Platt et al., 2010; Cao et al., 2011). Interestingly, the “East” subpopulation included accessions, mainly from Central Asia, with enhanced wound-induced RSA traits. In this case, genetic polymorphism may be strongly correlated with RSA traits because of demographic history, challenging the identification of the causal polymorphisms. Within the “West” subpopulation however, there was a clear longitudinal gradient of genetic polymorphisms, such as the accessions in Central Europe were also genetically close to those in the “East” subpopulation. Additionally, Nemrut-1 and Yeg-1 were included in the “East” subpopulation in our study, which is in agreement with a recently proposed migration route connecting Asia and Africa from the south (Brennan et al., 2014). Although it is well-known that population structure, among other effects, can complicate GWA studies in Arabidopsis (Filiault and Maloof, 2012), we reasoned that the studied traits showed broad phenotypic variation, globally as well as within the two subpopulations, that also exhibited continuous isolation by distance as observed earlier in this species (Platt et al., 2010). For example, some accessions from the “West” subpopulation, like Leo-1 and Pra-6, showed an enhanced root system after wounding, while other genetically and geographically close accessions (Cdm-0 and Qui-0, respectively) displayed a less complex RSA. It is well known that nutrients and other environmental signals in the soil might alter the RSA (Kellermeier et al., 2014). We thus speculate that the observed differences in wound-induced RSA traits might represent local adaptations to distinct ecological niches, and one of the environmental signals that might be involved is osmotic stress. Some accessions from the “East” subpopulation, such as Shigu-1 and Tamm-2, displayed lower RSA values than their close relatives. Hence, these contrasting accessions might be used as parents for QTL identification through conventional linkage association mapping in different soil stress conditions.

Through GWA mapping, we identified 162 SNP associations that significantly accounted for variation in wound-induced RSA traits located at 19 genomic regions, which were defined based primarily by non-synonymous SNPs. As expected by our trait correlation analyses, we found a clear overlap between three genomic regions associated for PR length and LR number (2/2′, 4/4′, and 5/5′). In all these cases, the causal polymorphism(s) might affect genes with pleiotropic effects on RSA. GWA mapping has facilitated the identification of the molecular variants underlying complex traits in crops, such as heterosis (Huang et al., 2016), grain size (Si et al., 2016), or drought resistance (Wang et al., 2016). In all these examples, hundreds of genetic variants were identified and candidate genes were assigned based on prior knowledge. In most cases, the genetic variation affected regulatory regions of candidate genes and functional validation using transgenic approaches were required for the functional validation of these genes.

Our results suggest that non-synonymous variation in the coding region of At4g01090 was significantly associated with wound-induced LR variation. At4g01090 encodes a hypothetical protein (DUF3133) of unknown function, which is expressed at higher levels in the endodermis of the elongation zone of the root and the mature xylem (Winter et al., 2007). Other ortholog genes encoding proteins with the DUF3133 domain are At4g01410, which has been annotated as a late embryogenesis abundant (LEA) protein, and *enhanced disease resistance 4* (*EDR4*), which modulates plant immunity by regulating clathrin heavy chain 2 (CHC2)-mediated vesicle trafficking (Wu et al., 2015). The protein encoded by At4g01410 has been found to interact with EDR4 (Mukhtar et al., 2011). One intriguing possibility is that these DUF3133-containing proteins might also interact with clathrin-coated vesicles during PIN-FORMED (PIN) endocytosis (Dhonukshe et al., 2007; Kitakura et al., 2011), which could then be directly linked to LR initiation (Ditengou et al., 2008). Additional experiments will be performed in our lab to confirm the involvement of DUF3133-containing proteins in clathrin-mediated PIN endocytosis during wound-induced LR formation.

At5g19710 encodes a histidine phosphotransfer protein belonging to the AHP bridge components of the His-Asp phosphorelay transduction pathway of CK signaling (Hwang et al., 2012). Five AHPs (AHP1-5) mediate the cytoplasmic-to-nuclear transduction of the CK signal by transferring the phosphoryl group from the CK receptors to nuclear type-B (positive) and type-A (negative) Arabidopsis response regulators (ARRs). AHP6 lacks the conserved His residue and negatively interferes with CK response (Moreira et al., 2013), most likely by competing with AHP1–5 for interaction with CK-activated receptors (Mahonen et al., 2006). The AHP protein encoded by the At5g19710 gene resembled AHP4. Based on loss-of-function analysis (Hutchison et al., 2006), a negative role of AHP4 for a subset of CK responses (i.e., LR formation) has also been proposed. Interestingly, At5g19710 is specifically expressed in LR cap cells in a low nitrogen environment while it was significantly downregulated by a short nitrate treatment (Gifford et al., 2007). Despite the local inhibitory role of CKs on LR initiation (Laplaze et al., 2007), CKs are essential components of the systemic signaling network leading to the enhancement of LR formation where nitrate is available (Ruffel et al., 2016). In Arabidopsis, the adaptive root response to nitrate depends on the NRT1.1/NPF6.3 transporter/sensor system (Bouguyon et al., 2016). NRT1.1 represses LR emergence at low nitrate concentration through its auxin transport activity that lowers auxin accumulation in the LR primordia (Bouguyon et al., 2016). An additional layer of regulation of systemic N signaling involves TCP20 (Guan et al., 2014). TCP20 is a transcription factor from the TEOSINTE BRANCHED1, CYCLOIDEA, and PCF (TCP) family that binds the promoters of type-A ARR5 and ARR7 at high nitrate levels and of NRT1.1 at low nitrate only (Guan et al., 2014). We speculate that the AHP protein encoded by At5g19710 might function as a negative regulator of a subset of CK responses in LR cap cells at low nitrate, leading to a net reduction of the number of competent sites for LR formation. Interestingly, cell-specific regulation of a transcriptional circuit including ARF8 and miR167 mediates LR outgrowth in response to nitrogen (Gifford et al., 2007). Additional experiments will be required to establishing a functional link between At5g19710-encoding AHP and the ARF8/miR167 circuit.

We found a statistically significant association between a non-synonymous SNP in the coding region of *K^+^ UPTAKE TRANSPORTER5 (KUP5)* that changes a Gln to His residue in a conserved transmembrane domain of the protein. Potassium is an essential element in plant growth as it affects osmotic regulation and cell water potential (Lebaudy et al., 2007). The Arabidopsis genome contains multigene families of potassium channels with distinct or redundant functions (Lebaudy et al., 2007), which might explain why the loss-of-function of kUP5 alone did not produce any effect on wound-induced RSA (this work). Consistently, loss-of-function mutations in three KUP family potassium efflux transporters, KUP6, 8 and 2, showed increased auxin responses and enhanced LR formation (Osakabe et al., 2013). As proposed earlier, these KUP transporters might coordinately control potassium homeostasis across root tissues, and the enhanced LR formation in the triple mutants might be caused by a local excess of potassium in the pericycle cells, resulting in enhanced LR formation due to its effect on cell cycle progression (Osakabe et al., 2013). We found an interesting epistatic interaction between the SNP polymorphisms in At4g33530 and At5g19710, which suggest a functional link between potassium uptake and CK signaling. CKs are fairly known to regulate uptake and metabolism of different nutrients: nitrogen, sulfate, phosphate, and iron (Brenner et al., 2012), but the roles of CKs in potassium signaling are poorly understood (Nam et al., 2012).

Through GWA mapping we have identified a number of significant non-synonymous polymorphisms that accounted for some of the variation found in wound-induced RSA. Our results highlighted new regulators of LR formation in Arabidopsis and the further dissection of the developmental mechanisms involved might help to understand the genetic basis of the natural variation of root plasticity.

## Author Contributions

JP-P was responsible for conceptualization and supervision and provided the funding acquisition. MJ and JP-P were responsible for methodology and performed the formal analysis. MJ, SI, and JP-P were involved in the investigation, writing of the original draft, and review and editing of the manuscript. AP was responsible for software development.

## Conflict of Interest Statement

The authors declare that the research was conducted in the absence of any commercial or financial relationships that could be construed as a potential conflict of interest.

## Footnotes

1 http://arabidopsis.info/

2 http://rsb.info.nih.gov/ij/

3 https://web.stanford.edu/group/pritchardlab/structure.html

4 http://gwas.gmi.oeaw.ac.at
