# Supplementary material for: A Genome-Wide Association Study Identifies New Loci Involved in Wound-Induced Lateral Root Formation in Arabidopsis thaliana
Source: Front Plant Sci. 2019 Mar 15;10:311. doi: 10.3389/fpls.2019.00311 (PMC6428781; doi:10.3389/fpls.2019.00311)
Supplement: Supplementary file 4 [file Table_4.DOCX]

% Read desired accessions and positions from input xls file

desired_accessions = xlsread('00_Dataset_Structure.xlsx','posADVRootNumber4DAC','B2:B121');

desired_positions = xlsread('00_Dataset_Structure.xlsx','posADVRootNumber4DAC','C2:C205979');

% Read hdf5 file

snp = hdf5read('all_chromosomes_binary_gzip.hdf5','snps');

all_positions = hdf5read('all_chromosomes_binary_gzip.hdf5','positions');

all_accessions_struct = hdf5read('all_chromosomes_binary_gzip.hdf5','accessions');

all_accessions = zeros(all_accessions_struct.length,1);

for i=1:all_accessions_struct.length

all_accessions(i,1) = str2num(all_accessions_struct(i).Data);

end

number_of_desired_accessions = length(desired_accessions);

number_of_desired_positions = length(desired_positions);

result = zeros(number_of_desired_accessions,number_of_desired_positions);

tic

stored_positions = zeros(number_of_desired_positions,1);

for i=1:number_of_desired_accessions

disp(100*(i-1)/number_of_desired_accessions);

current_desired_accession = desired_accessions(i);

row_of_desired_accession = find(all_accessions == current_desired_accession);

k = 1;

for j=1:length(all_positions)

current_position = all_positions(j);

is_desired_position = find(desired_positions == current_position);

if isempty(is_desired_position)

% Do nothing

else

result(i,k) = snp(row_of_desired_accession,j);

stored_positions(k,1) = current_position;

k = k + 1;

end

end

end

elapsed_time = toc
