# Supplementary material for: A Genome-Wide Association Study Identifies New Loci Involved in Wound-Induced Lateral Root Formation in Arabidopsis thaliana
Source: Front Plant Sci. 2019 Mar 15;10:311. doi: 10.3389/fpls.2019.00311 (PMC6428781; doi:10.3389/fpls.2019.00311)
Supplement: Supplementary file 7 [file Data_Sheet_1.PDF]

A Genome-Wide Association Study Identifies New Loci Required for Wound-Induced Lateral Root Formation in Arabidopsis thaliana

María Salud Justamante, Sergio Ibáñez, Adrián Peidró, José Manuel Pérez-Pérez

*** Correspondence:** José Manuel Pérez-Pérez (jmperez@umh.es)

1 Supplementary Figures and Tables

1.1 Supplementary Figures

Supplementary Figure S1

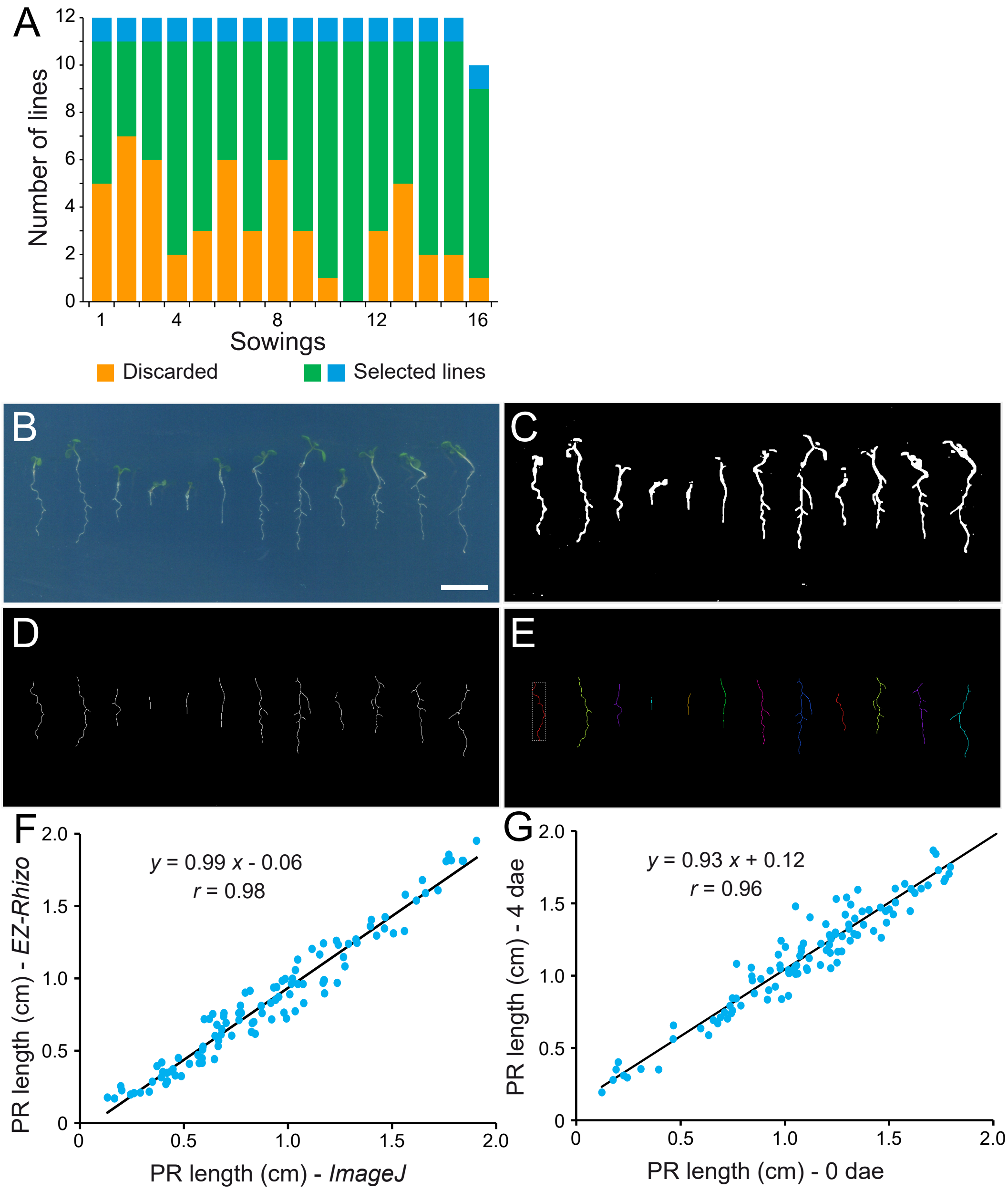

Supplementary Figure S1. Experimental procedure used for root analysis using EZ-Rhizo. (A) Number of accessions studied in each sowing. Those accessions with germination lower than 80% or with ambiguous marker information (orange bars) were discarded for further studies. Col–0 is shown in blue. (B) Petri dishes were individually scanned and cropped. Scale bar: 10 mm (C) Scans were converted into monochrome images using a default threshold and gaps were filled by using the 'Dilate' option. (D) Monochrome images were skeletonized and (E) roots within each image were semi-automatically detected as individual objects and the selected parameters were measured. (F) Scatter plot of PR lengths measured with EZ-Rhizo and ImageJ software. (G) Scatter plot of PR lengths measured at 0 and 4 dae.

Supplementary Figure S2

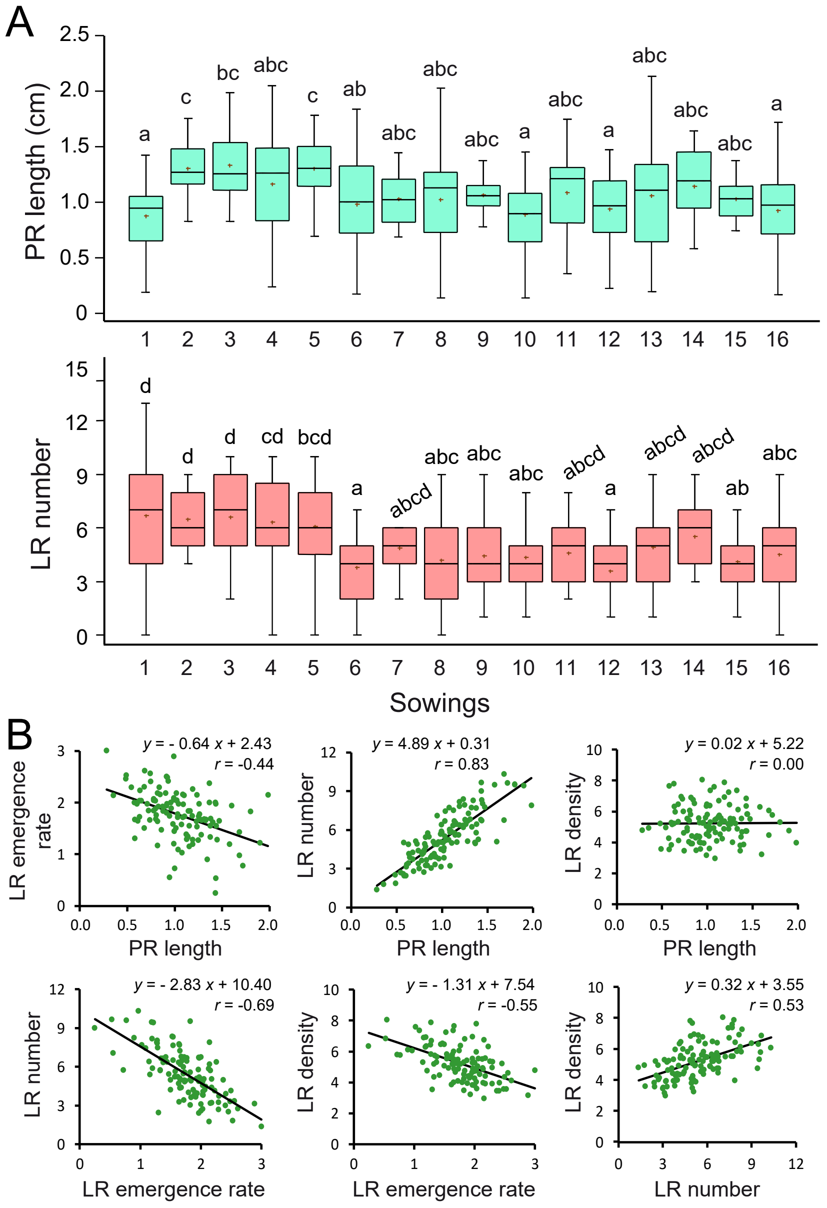

Supplementary Figure S2. Quantitative variation of the studied parameters. (A) Box-plots of PR length and LR number in Col–0 at 4 dae according to the different sowings. Letters indicate statistically significant differences (LSD; P-value < 0.05) between sowings. (B) Scatter plots of average values for the studied parameters in the different accessions.

Supplementary Figure S3

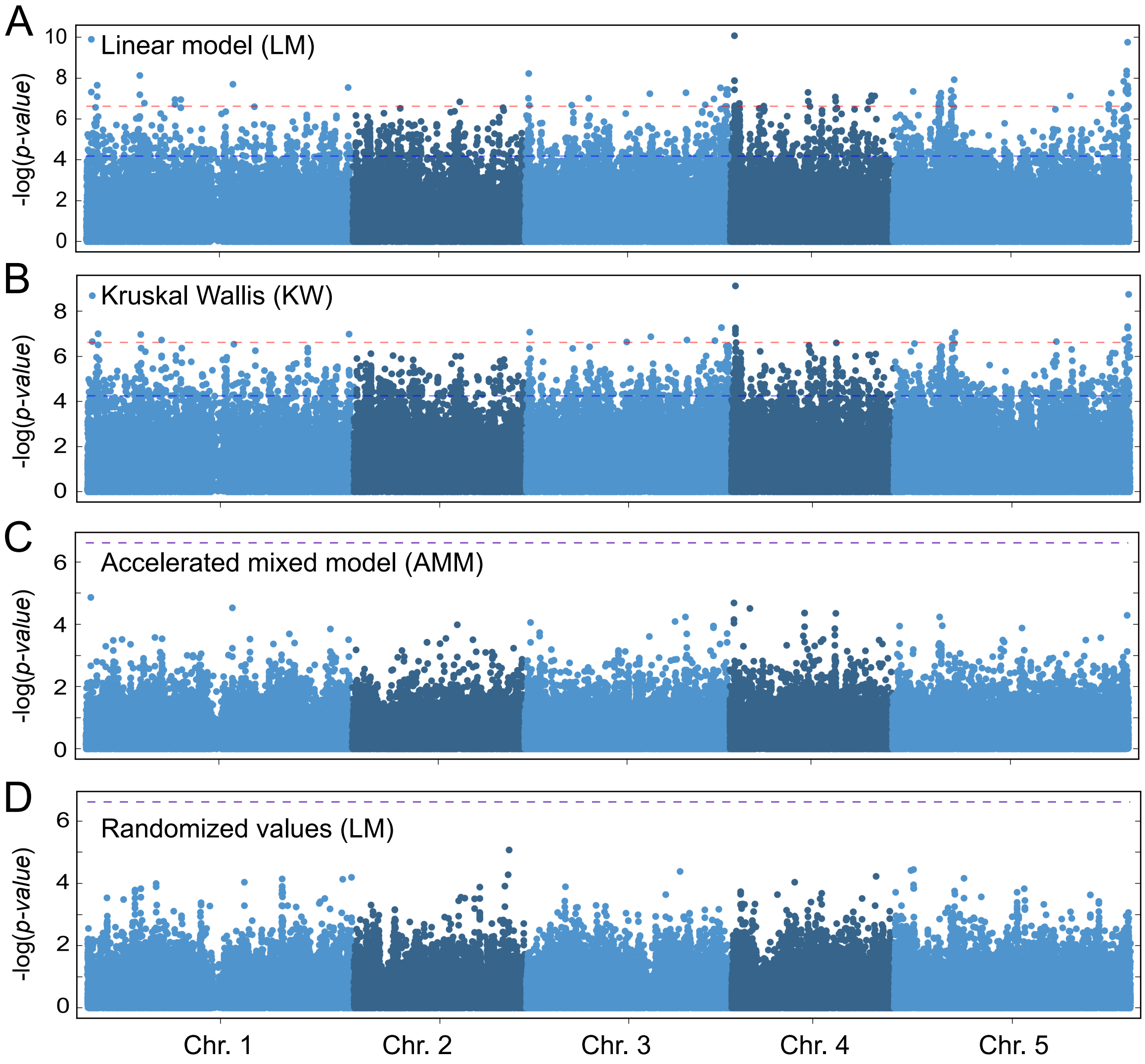

Supplementary Figure S3. Manhattan plots of associations between SNPs and relative LR number using different models. (A) Linear regression model (LM), (B) Kruskal-Wallis (KW) model and (C) accelerated mixed model (AMM). (D) Manhattan plot of association between SNPs and a randomized set of relative LR number values using a linear regression model. Dashed horizontal lines indicate threshold for significance in genome-wide association (GWA) mapping set at $-\log_{10}$(P-value)>6.5.

Supplemental Figure S4

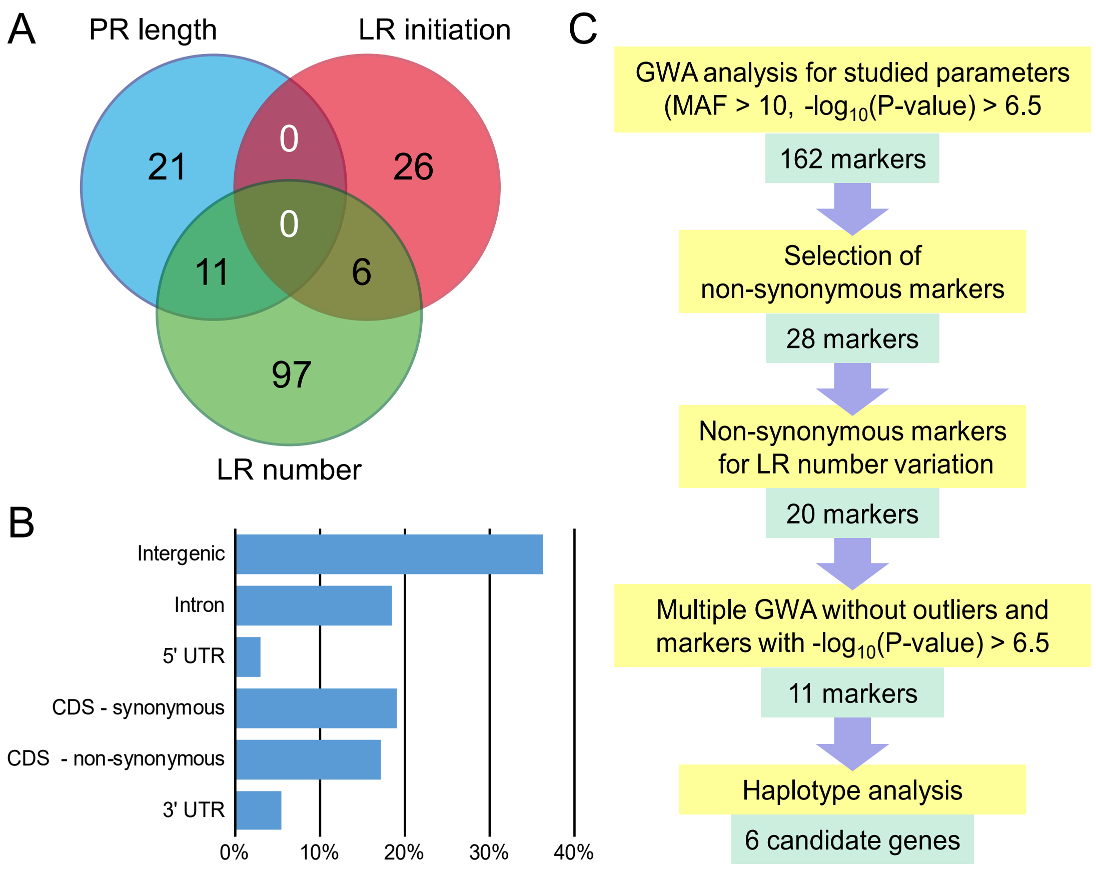

Supplementary Figure S4. Candidate gene selection. (A) Venn-diagram showing the number of statistically significant SNP markers identified from the studied parameters. The single SNP marker found for LR density is not included. (B) Frequency distribution of statistically significant SNP markers grouped according to the gene structural element they interrupt. CDS, coding sequence; UTR, upstream transcription start site. (C) Workflow chart followed for candidate SNP selection involved in LR number variation.

Supplemental Figure S5

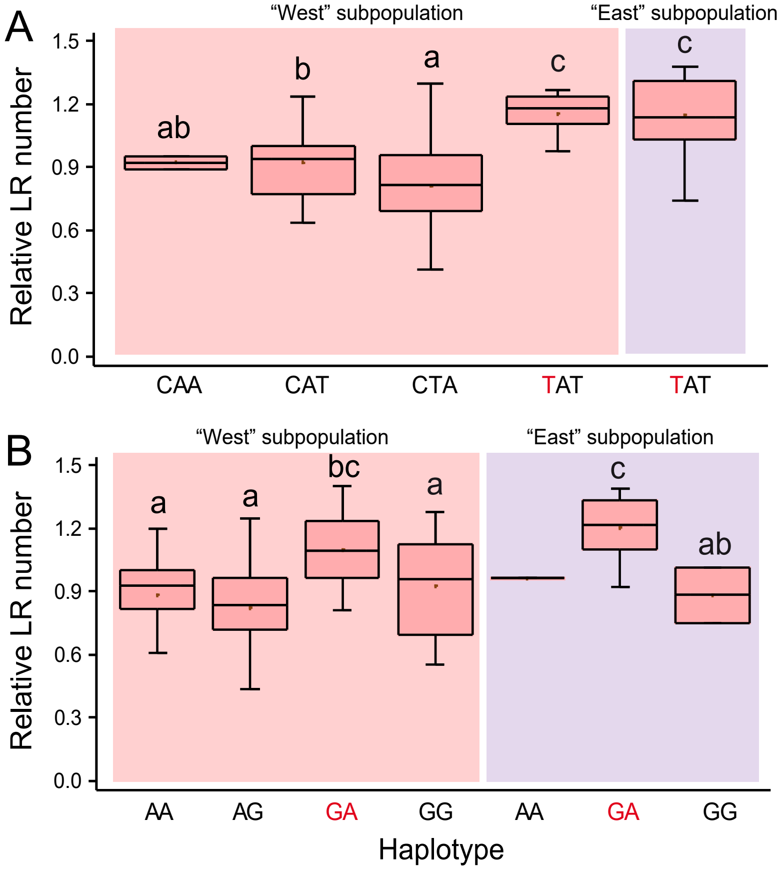

Supplementary Figure S5. Haplotype analysis for markers involved in LR number variation. In each subpopulation, accessions with the same haplotype for selected markers were grouped. Different letters indicate statistically significant differences (LSD; P-value < 0.05). (A) Non-synonymous SNP haplotypes for At4g01090. (B) Non-synonymous SNP haplotypes for At5g16220 and At5g19710. SNP positions associated with higher LR number values are depicted in red.

Supplementary Figure S6

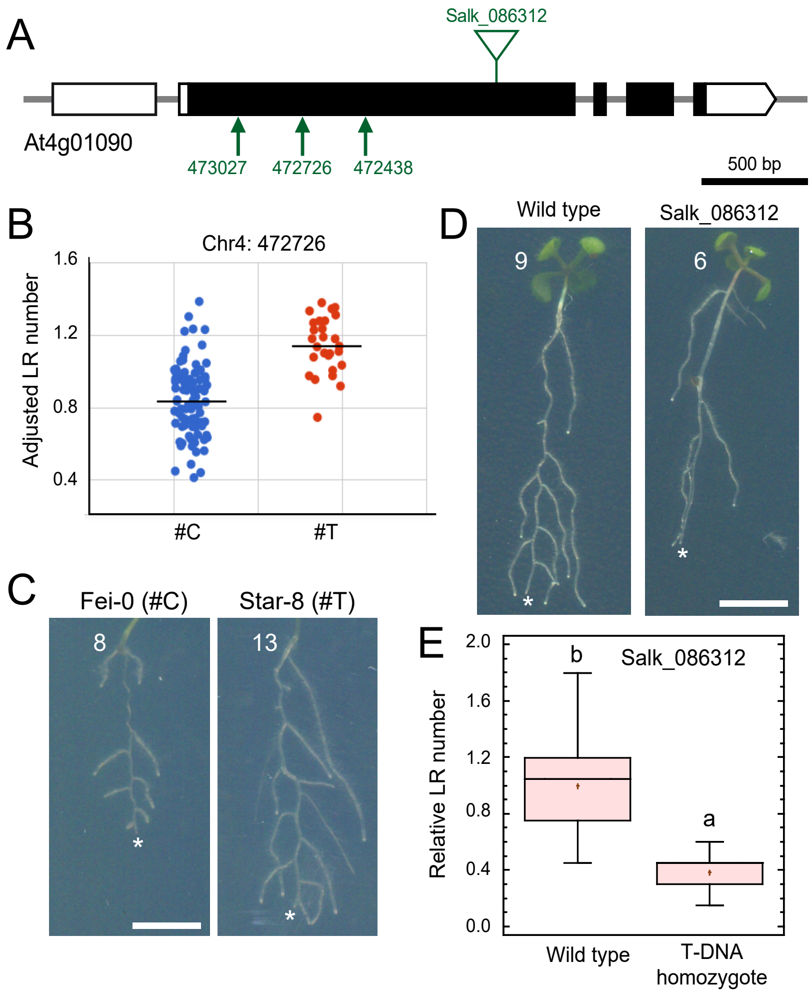

Supplementary Figure S6. Functional analysis of At4g01090 variation concerning LR number. (A) Gene structure of At4g01090. UTR regions and coding exons are represented by white and black boxes, respectively; introns are depicted as grey lines. The studied non-synonymous SNP markers (arrows) and annotated T-DNA insertions (triangles) are indicated in green. (B) Scatterplot of average LR number values used for GWA mapping, sorted by the alleles, C and T, at the Chr4:472726 position. (C) Some accessions of the studied haplotypes with contrasting LR number values. (D) Representative images of wild-type (left) and mutant (right) seedlings of indicated Salk lines at 4 dae. Asterisk marks the PR tip after excision and numbers refer to LR number at 4 dae. Scale bars: 5 mm. (E) Boxplot showing relative LR number values of wild type and homozygous T-DNA mutant plants from the Salk_086312 line. Different letters indicate significant differences (LSD, P-value<0.05).

Supplementary Figure S7

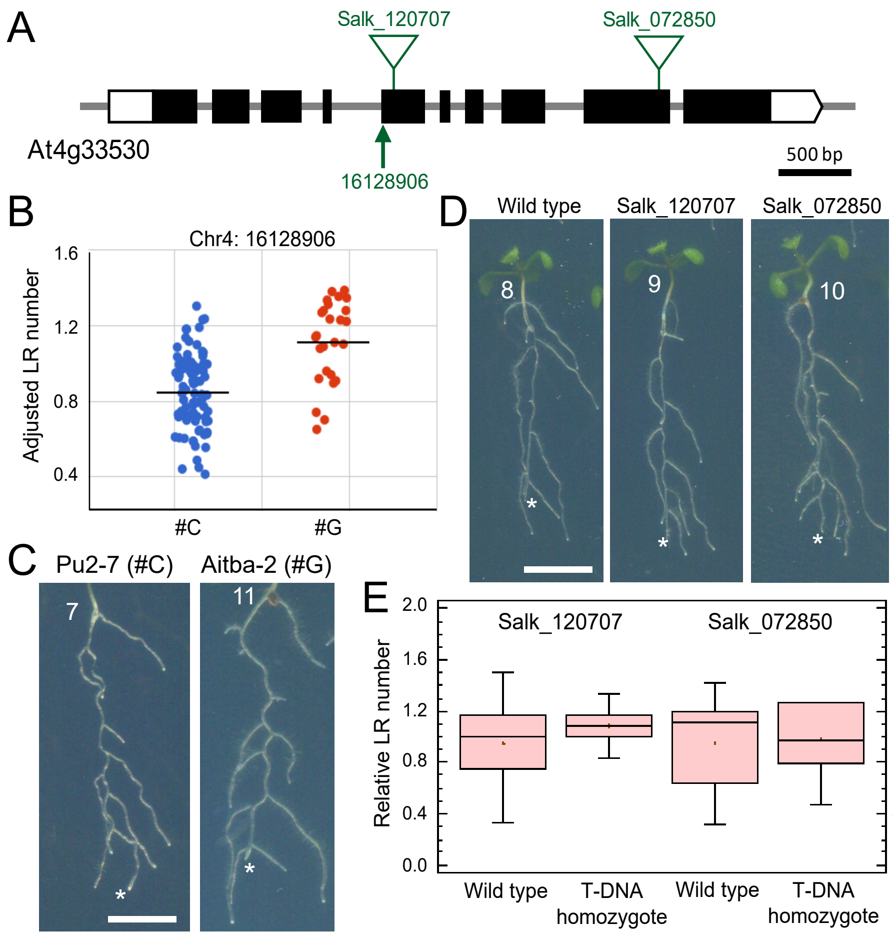

Supplementary Figure S7. Functional analysis of At4g33530 variation concerning LR number. (A) Gene structure of At4g33530. UTR regions and coding exons are represented by white and black boxes, respectively; introns are depicted as grey lines. The studied non-synonymous SNP markers (arrows) and annotated T-DNA insertions (triangles) are indicated in green. (B) Scatterplot of average LR number values used for GWA mapping, sorted by the alleles, C and G, at Chr4:16128906 position. (C) Some accessions of the studied haplotypes with contrasting LR number values. (D) Representative images of wild-type and T-DNA homozygous seedlings of indicated Salk lines at 4 dae. Asterisk marks the PR tip after excision and numbers refer to LR number at 4 dae. Scale bars: 5 mm. (E) Boxplot showing relative LR number values of wild type and homozygous T-DNA mutants of the indicated Salk lines.

Supplementary Figure S8

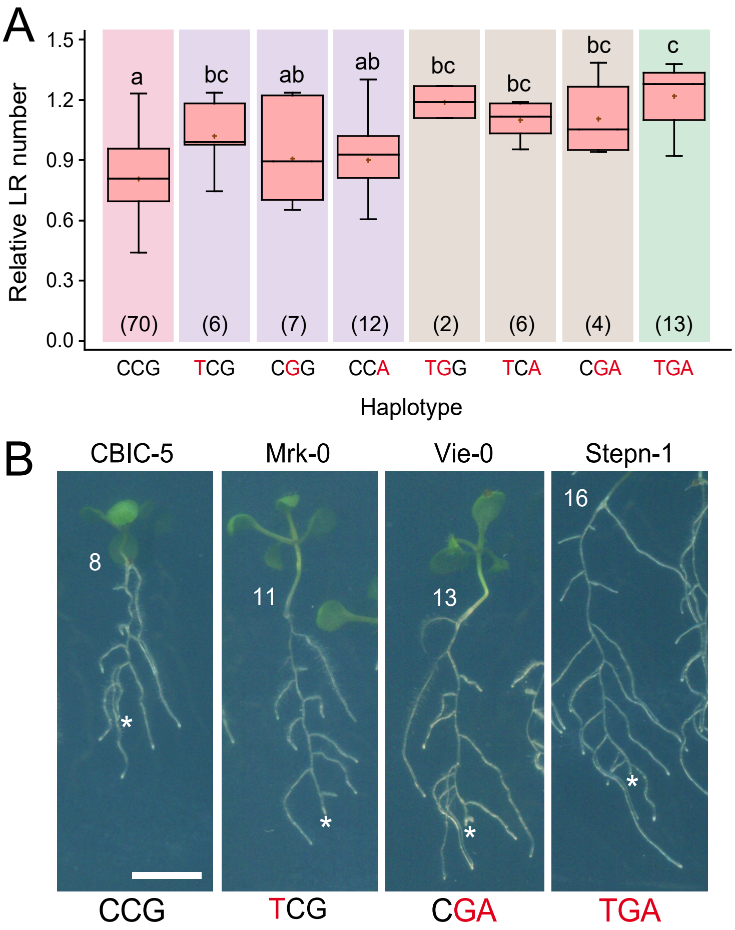

Supplementary Figure S8. Haplotype analysis for selected SNP markers in candidate genes involved in LR number variation. In each subpopulation, accessions with the same haplotype for selected markers were grouped. (A) Haplotypes for Chr4:472726, Chr4:16128906 and Chr5:6665363 markers. Different letters indicate statistically significant differences (LSD; P-value < 0.01). Numbers between parentheses refer to the number of accessions studied. Background colors refer to the number of allelic changes as regards the reference haplotype (CCG, red): one (purple), two (brown), three (green). (B) Some accessions with contrasting haplotypes correlated with wound-induced LR variation. Asterisk marks the PR tip after excision and numbers refer to LR number at 4 dae. Scale bars: 5 mm.

1.2 Supplementary Tables

Supplementary Table S1. ***Arabidopsis thaliana*** **accessions used in this study.** The accessions used for genome-wide association (GWA) mapping displayed high germination percentage (>80%) and unambiguous marker information (unique ID at the GWAPP web interface). N/A: not available at the time of experiment.

Supplementary Table S2. Oligonucleotides used in this study.

Supplementary Table S3. **Individual data values obtained in this work.**

Supplementary Table S4. **MATLAB script used for data selection and formatting.**

Supplementary Table S5. **Statistically significant SNP markers identified in this work.** Detailed information for each marker was obtained from the GWAPP web interface. GVE, genetic variance explained; MAC, minor allele count; MAF, minor allele frequency. We defined putative QTL (from 1 to 19) as indicated in the Results section.

Supplementary Table S6. **Haplotype diversity for statistically significant non-synonymous SNPs accounting for LR number.** See Supplementary Table S5 for legends.
